# Military Surgery in Turkey

**Published:** 1878-01

**Authors:** 


### Military Surgery in Turkey.

The following details will seem incredible to those who are not acquainted with the peculiar ways of the Turkish administration. An artillerist had his knee shattered at Sistova by the explosion of a shell, and after his wound had been temporarily dressed he was transported from the field of battle to Constantinople. In spite of his intense sufferings, he listened with the greatest interest to all the news from the seat of war. On his arrival in Constantinople, amputation was found to be necessary; but before the operation could be performed, permission had to be obtained from the ministry of war. This permission must always be obtained before an amputation can be performed in a Turkish hospital, and it not unfrequently happens that the patient dies before the civil functionaries have ceased deliberating on the demand of the surgeons. Fortunately for our artillerist, his case was pushed through with exceptionable rapidity, and the desired permit was given after a delay of only eight or ten days. The brave soldier, who had awaited the pleasure of the administration with the most exemplary patience, bore the operation with heroic courage; there is still hope that his life will be saved.—*The Medical Record.*

